# The HTIRDB: A resource containing a transcriptional atlas for 105 different tissues from each of seven species of domestic herbivore

**DOI:** 10.1002/imt2.267

**Published:** 2025-01-28

**Authors:** Luoyang Ding, Yifan Wang, Linna Zhang, Chengfang Luo, Feifan Wu, Yiming Huang, Yongkang Zhen, Ning Chen, Limin Wang, Li Song, Kelsey Pool, Dominique Blache, Shane K. Maloney, Dongxu Liu, Zhiquan Yang, Xiaoyan Huang, Chuang Li, Xiang Yu, Zhenbin Zhang, Yifei Chen, Chun Xue, Yalan Gu, Weidong Huang, Lu Yan, Wenjun Wei, Yusu Wang, Jinying Zhang, Yifan Zhang, Yiquan Sun, Rui Dai, Shengbo Wang, Xinle Zhao, Haodong Wang, Ping Zhou, Qing‐Yong Yang, Mengzhi Wang

**Affiliations:** ^1^ Laboratory of Metabolic Manipulation of Herbivorous Animal Nutrition, College of Animal Science and Technology Yangzhou University Yangzhou China; ^2^ State Key Laboratory of Sheep Genetic Improvement and Healthy Production Xinjiang Academy of Agricultural Reclamation Sciences Shihezi China; ^3^ Key Laboratory of Smart Farming for Agricultural Animals, Engineering Technology Research Center of Agricultural Big Data, College of Informatics Huazhong Agricultural University Wuhan China; ^4^ College of Life Science Guizhou University Guiyang China; ^5^ UWA Institute of Agriculture The University of Western Australia Perth Western Australia Australia

## Abstract

Here, we describe the Herbivore Transcriptome Integrated Resource Database (HTIRDB, https://yanglab.hzau.edu.cn/HTIRDB#/). The HTIRDB comprises the self‐generated transcriptomic data from 100 to 105 tissues from two female domestic herbivores from six species (cattle, donkey, goat, horse, rabbit, and sika deer) and two breeds of sheep, and an extra 28,710 related published datasets. The HTIRDB user‐friendly interface provides tools and functionalities that facilitate the exploration of gene expression between tissues and species. The tools for comparative transcriptomics can be used to identify housekeeping genes, tissue‐specific genes, species‐specific genes, and species‐conserved genes. To date, the HTIRDB is the most extensive transcriptome data resource for domestic herbivores that is freely available.

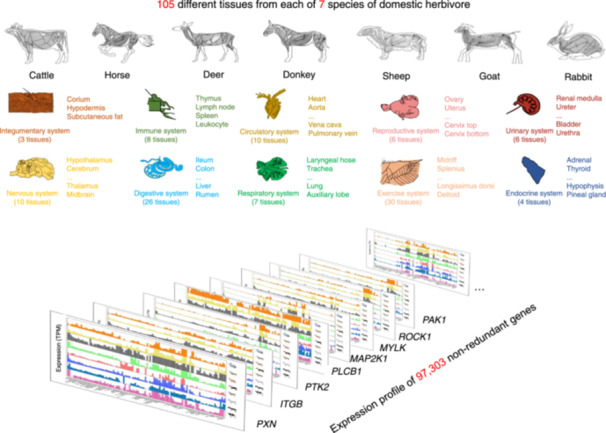

## CONFLICT OF INTEREST STATEMENT

The authors declare no conflicts of interest.

## ETHICS STATEMENT

All animal experiments were carried out in accordance with the ARRIVE guidelines, and the ethics application (No. RA202203046) was approved by the Animal Ethics Committee of Yangzhou University.

To the Editor,

Amongst herbivores, ruminants are essential to sustainable agriculture because they are well‐adapted to most environments and can utilize low‐quality feedstuff [1, 2]. To further integrate herbivores into sustainable agriculture practices, it is essential to understand how their genotypes interact with the environment to affect the phenotype. A cross‐species transcriptome atlas of gene expression in organs or tissues of different species of herbivore would help uncover the fundamental biological processes.

Several transcriptional atlases for some species of herbivore, such as cattle, sheep, donkeys, water buffalo, and goats, are available [3, 4, 5, 6]. However, the comparison of these datasets is difficult, if not impossible, because they are published in separate databases, and the sample collection, RNA sequencing, or data processing were not standardized across databases. Moreover, most of these databases used relatively small numbers of tissues and organs that are not always common to all databases.

**Figure 1:**
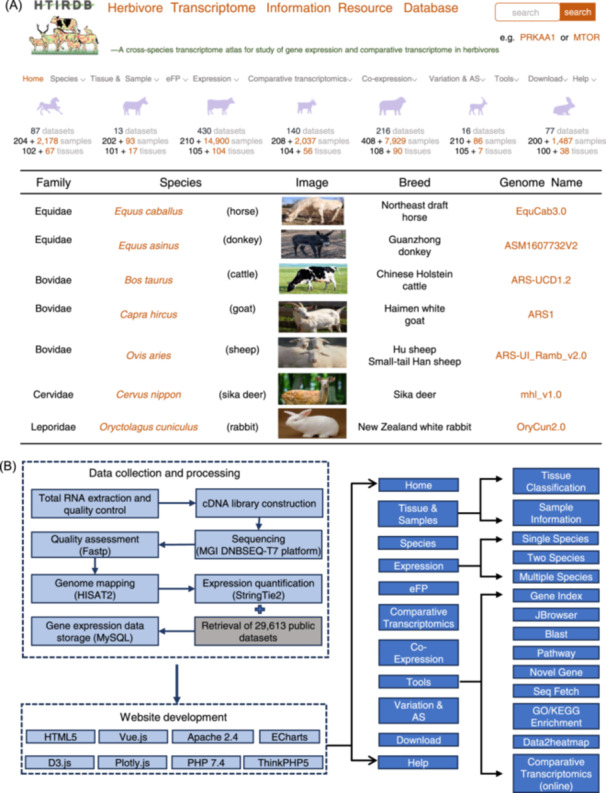
The features of the Herbivore Transcriptome Information Resource Database (HTIRDB). (A) The user interface of the HTIRDB. (B) A schematic overview of the data collection and construction of the HTIRDB.

Here we report the creation of the Herbivore Transcriptome Integrated Resource Database (HTIRDB, https://yanglab.hzau.edu.cn/HTIRDB#/), a public database that facilitates the comparison of gene expression profiles in a large number of tissues from seven species of domestic herbivore with high economic value (Figure 1A). The species includes cattle, deer, donkey, goat, horse, rabbit, and sheep. Both Hu and small‐tail Han sheep were included because of their economic relevance in China. The HTIRDB hosts the self‐generated transcriptomic data of 100–105 tissues from two females of each species that was collected using a standard protocol [7], and the published transcriptomic datasets that have close relationship to these herbivores. In addition, the HTIRDB provides nine bioinformatics tools, all publicly available, to facilitate the use of the database by biologists.

## RESULTS

### An overview of the HTIRDB

On the homepage of the HTIRDB (Figure 1A), the “Species” and “Tissue and Samples” panels link to information on the species and tissue samples. The “Sample Information” module displays the anatomical distribution and the tissue coding system as tables or diagrams. The HTIRDB offers four functionalities for data retrieval and visualization: electronic fluorescent pictographic (eFP), expression, comparative transcriptomics, and co‐expression analysis. The HTIRDB provides a suite of nine bioinformatic tools (Figure 1B).

### Data summary

The HTIRDB hosts 30,352 datasets, comprising 1642 self‐generated RNA‐sequences (RNA‐Seq) datasets (mapping rate 93.9 ± 4.0%, Table S1), and 28,710 RNA‐Seq datasets (mapping rate 94.8 ± 5.5%, Table S2) from published databases, contributing to approximately 638 billion high‐quality sequencing reads.

### A vivid visualization of gene expression using eFP

**Figure 2:**
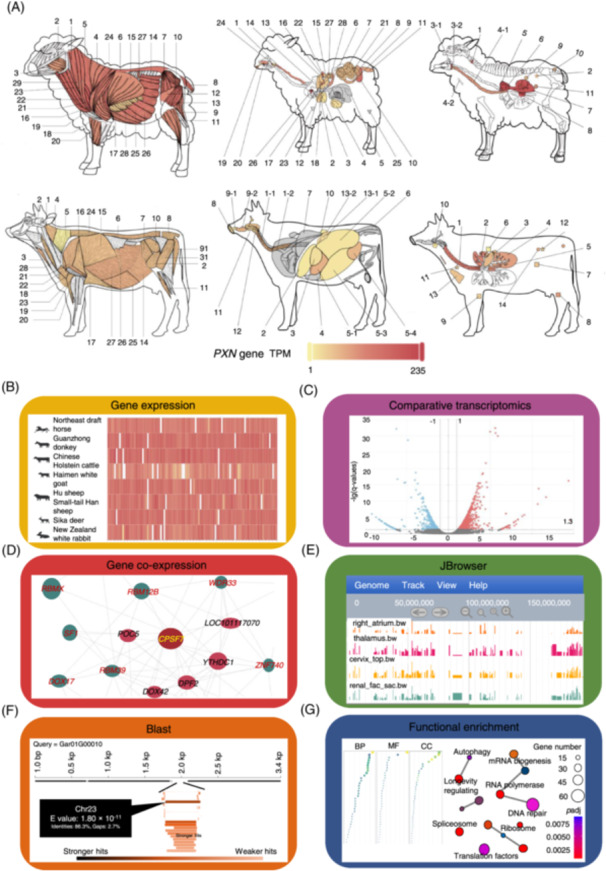
The resources and tools available in the Herbivore Transcriptome Information Resource Database. (A) Examples of electronic fluorescent pictograph output for the expression of *paxillin* gene in sheep and cattle. Functions and tools that are available for the database, including (B) gene expression, (C) comparative transcriptomics, (D) gene co‐expression, (E) JBrowser, (F) Blast, and (G) functional enrichment using Gene Ontology, and Kyoto Encyclopedia of Genes and Genomes enrichment.

The eFP panel was built using Python Imaging Library Build (www.python.org). The corresponding anatomical location of the expression of a gene is presented on a pictographic representation of each animal with different colors (Figure 2A). The “Single Species” module enables a user to visualize the absolute or relative expression of any target gene in different tissues within a species. Additionally, users can compare a ratio of the relative expression of a primary gene to a reference gene in different tissues within the same species. The “Two Species” module compares the relative expression of any two genes in different tissues between two species or breeds. The “Multiple Species” module compares the relative expression of a target gene in one species to the relative expression of the same gene in any of the other species or breeds.

### Expression

The “Expression” panel, comprising three modules, provides a comprehensive platform to access expression data on mRNA or lncRNA (Figure 2B). The “Multi‐genes in Single Species” module analyses the expression of single or multiple genes between different tissues within the same species/breed or between different tissues from different species/breeds. The “LncRNA Expression Profile” module provides queries for the expression of lncRNA between different tissues.

The relative expression can be visualized as a table, heatmap, broken line chart, or box plot. The “Expression” panel offers a unique module called “Query by Sample,” which presents the available datasets in a table so that a user can select datasets across different breeds and tissues.

### Comparative transcriptomics

The panel “Comparative Transcriptomics” includes two modules that allow for comparison of expression in “Single Species” or between “Multiple Species.” The expression of selected genes can be compared in the same species between any two tissues or in one tissue between any two species (Figure 2C). The user can identify differentially expressed genes (DEGs) between any two tissues in the same species or between one tissue in any two species. The outputs are visualized with volcano plots, tables, and bar plots that report the 20 genes with the largest differences in expression. The tissue‐specific genes (TSGs) and housekeeping genes (HKGs) can be retrieved in the single species section, while species‐specific genes (SSGs) and species‐conserved expressed genes (SCGs) can be identified in the “Multiple Species” module using system defaults or user‐defined parameters [8].

### Co‐expression

The “Co‐expression” panel creates co‐expression networks by visualizing genes with similar expression patterns in all tissues in any single species/breed (Figure 2D). Once the genes of interest, the species, and the parameters are selected, a visual representation of the gene interactions is generated together with a table of Pearson's correlation coefficients.

### Variation and alternative splicing

The “Variation and Alternative Splicing” panel provides queries for genomic variation within a specified genomic region of the target genes using a gene structure plot, and the annotation details of those variations with a table and a pie chart. The “Alternative Splicing” model provides options to visualize the types and index values of splicing events for the target genes across different tissues.

### General tools

The “Tools” panel provides additional bioinformatics tools. For example, the “JBrowser” viewer generates a graphical overview of the newly generated RNA‐seq datasets to illustrate the expression profile of samples relative to the entire reference sequences (Figure 2E). The “Blast” tool enables users to interrogate and download reference sequences, including genome sequences, coding sequences, and protein sequences of specific genes (Figure 2F). The “Gene Ontology and Kyoto Encyclopedia of Genes and Genomes (GO&KEGG) Enrichment” function allows analysis of gene function to explore the biological roles and pathways associated with target genes (Figure 2G). The “Pathway” interface integrates the KEGG pathways and visualizes the genes involved in a specific pathway for a given species. A “Novel Gene” tool in “Download” identifies, compares, and visualizes the expression of new transcriptome data.

## DISCUSSION

The main goal of the HTIRDB was a research focus on the transcriptomics of domestic herbivores. To date, the HTIRDB provides the largest transcriptomic data resource for domestic herbivores. The website was created to offer access to the transcriptomic data of 100–105 tissues that was obtained with the same sampling protocol, from seven species and two breeds of domestic herbivore and to facilitate the exploration of those datasets (as shown in Figure S1 in case study 1 in the Supporting Information).

Compared to published databases on animal transcriptome, the HTIRDB can be used primarily to further identify the genetic foundations of economically significant traits and novel pathways for genetic improvement (as shown in case study 2 in the Supporting Information, Figure S2 and Tables S3–S5). Secondarily, the HTIRDB could facilitate further understanding of the evolutionary processes that have taken place in herbivores during their domestication and the interaction between genetics and environment on the expression of similar and different phenotypes (see case study 3 in the Supporting Information, Figure S3 and Table S6). The bioinformatic tools and functionalities are user‐friendly. Users can easily study patterns of gene expression, functional annotations, and comparative analyses within and between a range of tissues of domestic herbivores. The HTIRDB is the first database that includes eFP to visualize the expression levels of any gene in the entire body of herbivores. The hierarchical coding system for all tissue samples facilitates both comprehension of the datasets and their processing while avoiding linguistic ambiguity.

It's important to acknowledge that the current iteration of HTIRDB has some limitations. First, the public RNA‐Seq focuses mainly on tissues or the biological systems and species (cattle and sheep) of herbivores that have more economic benefits and that limits the interpretation of the transcriptomic data available in the HTIRDB. Second, HTIRDB lacks data on important systems and tissues, such as male reproduction. Third, the expression profile of some genes might not be in the HTIRDB because these genes were not included in reference genomes used for gene annotation.

The HTIRDB is a living platform that will include transcriptomics data from other species or breeds as they become available. If funding permits, new tools and databases will be added to the HTIRDB to ensure its relevance over time.

## METHODS

### Data collection and processing

The data stored in the HTIRDB were obtained as follows.

First, we generated data using transcriptome sequencing from between 100 and 105 tissue samples that were dissected from two healthy juvenile female individuals of seven species of herbivores, including two breeds of sheep (details of experimental animals are shown in Table S7). Sample collection, total RNA extraction, and transcriptomic sequencing were described in a previous publication [7].

Second, we mined published RNA‐Seqs that were generated with ILLUMINA (read length > 50 bp, sequencing depth (clean read number) > 2 million) from the National Center for Biotechnology Information Sequence Read Archive database (NCBI SRA) for each of the seven species [9, 10, 11, 12, 13]. Public RNA‐Seq of species closely related to the seven species, such as buffalo, yak, cattle‐yak, and hares, were also mined for further analysis (the codes for grouping tissue samples are listed in Table S8). The codes for quality control and data processing are reported in Table S9.

The gene expression data in different tissues in each of the seven species of herbivores were calculated with either the self‐generated transcriptomic data or a mixture of the self‐generated and the published transcriptomic data (Figure 1B).

### Construction of database

The data on gene expression for all the RNA‐Seq were stored in MySQL (https://www.mysql.com). Apache 2.4 (http://www.apache.org), PHP 7.4 (http://www.php.net), and ThinkPHP 5.0 (http://www.thinkphp.cn/) were used to create user access to the content of the database. The web pages were constructed using HTML5 and rendered using Element (https://element.eleme.cn/) based on Vue 2.0 (https://cn.vuejs.org). The JavaScript framework Vue.js was used to improve the user experience. Plotly.js, D3.js, and ECharts (https://echarts.apache.org/) were used to visualize the level of gene expression and results of the comparative transcriptomics. A combination of these tools enables data visualizations, such as heatmaps, line charts, boxplots, volcano plots, and histograms (Figure 1B).

### Construction of basic bioinformatic tools

Nine tools were integrated into the webpage to enhance the functionality and utility of the HTIRDB. Diamond v0.9.14 facilitates the acquisition of gene positions across the eight reference genomes [14]. Jbrowser provides structural and comparative genomics visualization [15]. Blast through Sequenceserver enables sequence alignment [8]. The Sequence Fetch tool, powered by Samtools v1.15 and bedtools [16], allows easy retrieval of sequences like genomic DNA, coding DNA, or protein sequences for target genes. We constructed a GO/KEGG enrichment analysis tool utilizing ClusterProfiler [17], eggNOG‐mapper v2 and eggNOG 5.0 [18], and DIAMOND [19] to support gene functional analysis. Data2heatmap using Echarts (https://echarts.apache.org/) provides users with an intuitive and interactive heatmap representation of their gene expression data.

### Comparative transcriptomic analysis

To compare the levels of gene expression between tissues/species, we retained genes with more than ten reads that were mapped in at least two samples. Next, we used the R package “DESeq.2” to identify DEGs using two cut‐off criteria: (i) A false discovery rate (FDR) < 0.05, and (ii) |log2 fold change| > 1 [20]. An expression specificity index τ was calculated to identify TSGs, HKGs, SSGs, and SCGs (details are reported in Supporting Information).

### Identification of genomic variation and alternative splicing

Alternative splicing events were identified using rMATs on BAM files from RNA‐Seq data. Single nucleotide polymorphisms and insertion‐deletion were called and annotated using GATK's and snpEff (v5.2a), respectively (details are reported in Supporting Information).

### Identification of lncRNAs and novel transcripts

StringTie (v2.1.4) was employed to assemble transcripts of BAM files from the self‐generated RNA‐Seq. A combination of TACO (v0.7.3), gffcompare (v0.12.6), gffread, Rfam (v14.9), blastn (v2.9.0+), CPC2 (v1.01), PLEK (v2.1), CNCI, and TransDecoder (v5.5.0) were used subsequently to identify lncRNAs and novel transcripts (details are reported in Supporting Information).

### Construction of a gene co‐expression network

A co‐expression network was generated using Cytoscape (https://js.cytoscape.org/). The co‐expression network was constructed by computing the Pearson correlation coefficient between each pair of genes.

## AUTHOR CONTRIBUTIONS

**Luoyang Ding** and **Yifan Wang**: Methodology; investigation; writing—original draft; writing—review and editing. **Linna Zhang** and **Chengfang Luo**: Formal analysis; data curation; software; writing—review and editing. **Feifan Wu**: Investigation; methodology; writing—review and editing. **Yiming Huang**: Software; data curation. **Yongkang Zhen** and **Kelsey Pool**: Methodology; writing—review and editing. **Ning Chen**: Investigation; methodology; project administration. **Limin Wang**, **Li Song**, **Chuang Li**, **Xiang Yu**, **Zhenbin Zhang**, **Chun Xue**, **Yalan Gu**, **Weidong Huang**, **Lu Yan**, **Wenjun Wei**, **Yusu Wang**, **Jinying Zhang**, **Yifan Zhang**, and **Yiquan Sun**: Investigation; methodology. **Dominique Blache**: Writing—review and editing; investigation. **Shane K. Maloney**: Writing—review and editing; methodology. **Dongxu Liu**: Formal analysis; software; data curation. **Zhiquan Yang**: Formal analysis; software; visualization. **Xiaoyan Huang**: Software. **Rui Dai**: Formal analysis. **Shengbo Wang** and **Xinle Zhao**: Validation; visualization. **Haodong Wang**: Formal analysis; data curation. **Ping Zhou** and **Mengzhi Wang**: Conceptualization; funding acquisition; supervision; writing—review and editing; investigation. **Qing‐Yong Yang**: Conceptualization; supervision; funding acquisition; writing—review and editing; formal analysis; software.

## Supporting information


**Figure S1.** An example showing the expression of the *paxillin* gene between the seven species.
**Figure S2.** Comparison of gene expression in the *longissimus dorsi* of sheep and goats.
**Figure S3.** An overview of the pattern of expression and functional enrichments of housekeeping genes for each of eight breeds of herbivore.

**Table S1.** An overview of the self‐generated RNA‐Seq.
**Table S2.** An overview of the public RNA‐Seq.
**Table S3.** Genes differentially expressed in the *longissimus dorsi* between small‐tail Han sheep and Haimen white goat.
**Table S4.** Genes differentially expressed in the *longissimus dorsi* between small‐tail Han sheep and Hu sheep.
**Table S5.** Genes differentially expressed in the *longissimus dorsi* between Hu sheep and Haimen white goat.
**Table S6.** House‐keeping genes for each of eight breeds of herbivore.
**Table S7.** Details of experimental animals that were used for self‐generated RNA‐Seq.
**Table S8.** List of organ and system codes.
**Table S9.** Codes that were used for database construction.

## Data Availability

The HTIRDB is an open database, with all RNA‐Seq datasets, freely available at https://yanglab.hzau.edu.cn/HTIRDB#/download. The raw self‐generated RNA‐Sequence reads have been deposited in the NCBI SRA database (https://www.ncbi.nlm.nih.gov/bioproject/?term=PRJNA1017964). Supplementary materials (methods, figures, tables, graphical abstract, slides, videos, Chinese translated version, and update materials) may be found in the online DOI or iMeta Science http://www.imeta.science/.
