# Supplementary material for: The HTIRDB: A resource containing a transcriptional atlas for 105 different tissues from each of seven species of domestic herbivore
Source: Imeta. 2025 Jan 28;4(1):e267. doi: 10.1002/imt2.267 (PMC11865344; doi:10.1002/imt2.267)
Supplement: Supplementary file 1 — Figure S1. An example showing the expression of the paxillin gene between the seven species. Figure S2. Comparison of gene expression in the longissimus dorsi of sheep and goats. Figure S3. An overview of the pattern of expression and functional enrichments of housekeeping genes for each of eight breeds of herbivore. [file IMT2-4-e267-s002.docx]

Supporting information to

**The HTIRDB: a resource containing a transcriptional atlas for 105 different tissues from each of seven species of domestic herbivore**

**Running title**: The transcriptional atlas for herbivores at cross-tissues and cross-species levels

Luoyang Ding^1,5#^, Yifan Wang^1,2,4#^, Linna Zhang^2,^^3#^, Chengfang Luo^3#^, Feifan Wu^1#^, Yiming Huang^2,3#^, Yongkang Zhen^1,2^, Ning Chen^2^, Limin Wang^2^, Li Song^4^, Kelsey Pool^5^, Dominique Blache^1,5^, Shane K Maloney^5^, Dongxu Liu^3^, Zhiquan Yang^3^, Xiaoyan Huang^3^, Chuang Li^1^, Xiang Yu^1^, Zhenbin Zhang^1^, Yifei Chen^1^, Chun Xue^1^, Yalan Gu^1^, Weidong Huang^1^, Lu Yan^1^, Wenjun Wei^1^, Yusu Wang^1^, Jinying Zhang^1^, Yifan Zhang^1^, Yiquan Sun^1^, Rui Dai^3^, Shengbo Wang^3^, Xinle Zhao^3^, Haodong Wang^3^, Ping Zhou^2^*, Qing-Yong Yang^2,3^*, Mengzhi Wang^1^*

^1^ Laboratory of Metabolic Manipulation of Herbivorous Animal Nutrition, College of Animal Science and Technology, Yangzhou University, Yangzhou 225009, China

^2^ State Key Laboratory of Sheep Genetic Improvement and Healthy Production, Xinjiang Academy of Agricultural Reclamation Sciences, Shihezi 832000, China

^3^ Key Laboratory of Smart Farming for Agricultural Animals, Engineering Technology Research Center of Agricultural Big Data, College of Informatics, Huazhong Agricultural University, Wuhan 430070, China

^4^ College of Life Science, Guizhou University, Guiyang 550025, China

^5^ UWA Institute of Agriculture, The University of Western Australia, Perth WA 6009, Australia

^#^ These authors contributed equally: Luoyang Ding, Yifan Wang, Linna Zhang, Chengfang Luo, Feifan Wu, and Yiming Huang.

^*^Correspondence: [zhpxqf@163.com](mailto:zhpxqf@163.com) (Ping Zhou); [yqy@mail.hzau.edu.cn](mailto:yqy@mail.hzau.edu.cn) (Qing-Yong Yang); [mzwang@yzu.edu.cn](mailto:mzwang@yzu.edu.cn) (Mengzhi Wang)

**SUPPLEMENTARY RESULTS**

**Case studies for application of the HTIRDB**

Case Study 1: Browsing and visualizing expression the *paxillin* (*PXN*) gene

We first interrogate the database to compare the relative expression of the gene that encodes for PXN in different tissues in all of the species. The module “single gene in multi-species” under “Expression” was used. The translated product of the *PXN* gene is a protein that recruits multiple intermediates of specific signalling pathways and facilitates the integration and processing of adhesion- and growth factor-related signals [1]. The level of gene expression varied between tissues, with relatively higher expression in the thymus, deltoid muscle, lobus inferior pulmonis dextri, auxiliary lobe, lobus medius pulmonis dextri, and lobus superior pulmonis, and lower expression in the medulla oblongata, hypothalamus, pons, hypophysis, and spinal cord. The module provides both a heatmap and a CSV file of the expression data that can be used to generate graphs using Origin (Figure S1).

Case study 2: Identification of differentially expressed genes (DEGs) in the *longissimus dorsi* in sheep and goat

We analysed differentially expressed genes (DEGs) in the *longissimus dorsi* between Small-tail Han sheep, Hu sheep, and Haimen white goat. A total of 4788 DEGs were identified between Small-tail Han sheep and Haimen white goat, including 2396 genes that were expressed more, and 2392 genes that were expressed less, in the goat than the sheep (Figure S2A, details of DFGs reported in Table S6). The GO & KEGG enrichment function revealed that the DEGs that were expressed more in the goat were mainly enriched in hypertrophic cardiomyopathy, dilated cardiomyopathy, calcium signalling pathway, ion channels, and cardiac muscle contraction pathways. The DEGs that were expressed less in the goat were mainly enriched in ribosome biogenesis, cytokine receptors, MAPK signalling pathway, and TNF signalling pathway (Figure S2B). A similar number of DEGs (4119 genes with details in Table S7) in *longissimus dorsi* were differentially expressed between the Hu sheep and Haimen white goats (Figure S2C). The KEGG enrichment analysis for the genes that were expressed more and less between Hu sheep and Haimen white goats revealed similar pathways to the comparison of the Han sheep and goat (Figure S2D). A smaller number of DEGs in *longissimus dorsi* were identified between the two breeds of sheep (Hu and Small-tail Han; 745 genes that are detailed in Table S8) and were mainly enriched in ion channels, complement and coagulation cascades, and PPAR signalling pathway (Figure S2E, F). The results presented above would help to investigate the mechanisms that underlie muscle development in sheep and goats and advance our understanding of the molecular processes that are involved in muscle growth and development in these herbivores.

Case study 3: Identification of house-keeping genes (HKGs)

House-keeping genes were defined as genes that were constitutively expressed in more than 80% of tissue samples in a species/breed. Given that expression profile, those genes are thought to be vital for the maintenance of essential biological processes and cellular functions [2]. Under the panel “Comparative transcriptomics” the HKG function identified 732 HKGs in Northeast draft horses, 801 in Guanzhong donkeys, 475 in Chinese Holstein cattle, 642 in Haimen white goats, 1043 in Hu sheep, 239 in Small-tail Han sheep, 570 in sika deer, and 452 in New Zealand white rabbits (Figure S3A, details of HKGs are in Table S9). Nineteen genes reached the HKG threshold across all seven species and included mitochondrial E3 ubiquitin protein ligase 1 (*MUL1*), signal recognition particle 68 (*SRP68*), proteasome 20S subunit alpha 3 (*PSMA3*), and poly(rC) binding protein 1 (*PCBP1*) (Figure S3B).

We further investigated the large number of HKGs in Hu sheep. A GO enrichment showed that the 1043 HKGs in Hu sheep were enriched mainly in mitochondrial gene expression, mitochondrial translation, translational termination for biological processes, catalytic activity acting on tRNA, catalytic activity acting on nucleic acid, and ribonucleoprotein complex binding for molecular function, as well as mitochondrial matrix, organellar ribosome, and mitochondrial ribosome for cellular component (Figure S3C). The KEGG enrichment indicated that the HKGs in Hu sheep were enriched in spliceosome, proteasome, mitochondrial biogenesis, translation factors, transcription machinery, and ribosome biogenesis (Figure S3D).

**SUPPLEMENTARY METHODS**

**Parameters for the definition of tissue-specific gene (TSGs), housekeeping genes (HKGs), specific species genes (SSGs), and species conserved genes (SCGs)**

A expression specificity index τ was calculated using the following formula to categorise genes as either tissue-specific or housekeeping genes [3].

$$\tau=\frac{\sum_{i=1}^{N} (1-x_{i})}{N-1}$$

where *N* was the number of tissues and $x_{i}$ was the expression of a given gene in the *i*’th sample which was normalized to the maximum expression level among all samples.

Within the database, a gene was initially classified as TSGs when its τ value exceeded 0.95 and the expression value of this gene in at least one tissue was greater than 1. Genes expressed in 80% of tissues and with both τ and coefficient of variation (CV) values of less than 0.5 were classified as HKGs. The effect of varying those cut-off values for τ and CV can be explored via a manual adjustment by the user. Genes were classified as SSGs if they were common among all seven reference genomes and present in tissue samples from all eight species/breeds, and a τ value greater than 0.95, and the expression value of the gene in at least one species was greater than 1. Genes categorized as SCGs must be expressed in all species studied and have both a τ value and a CV of expression of less than 0.5.

**Identification of lncRNAs and novel transcripts**

StringTie (v2.1.4) was employed to assemble transcripts of BAM files from the self-generated RNA-Seq data [4]. Assembled transcripts with low read coverage were filtered using the parameters “-c 5 -j 3”. The retained transcripts were subsequently merged using TACO (v0.7.3) [5]. To annotate these merged transcripts, they were compared to reference transcript annotations using gffcompare (v0.12.6) [6], and their sequences were extracted from the merged GTF file using gffread [7]. To exclude known mRNAs, only the transcripts with the class codes “u” or “x.” were retained. Low-quality transcripts were further filtered out based on the following criteria: 1) Transcripts shorter than 200 bp; 2) Transcripts with undefined strand information; 3) Transcripts containing “N” bases in their sequences. Subsequently, the filtered transcripts were aligned against the Rfam (v14.9) database using blastn (v2.9.0+) to remove housekeeping non-coding RNAs [8,9]. The coding potential of the remaining transcripts was predicted using three tools: CPC2 (v1.01), PLEK (v2.1), and CNCI [10,11]. For PLEK and CNCI, the “-m ve” parameter was applied to restrict predictions to vertebrates. Transcripts identified as non-coding by all three tools were classified as novel lncRNAs, while those classified as coding by at least two tools were considered as candidate mRNAs. To refine the set of candidate mRNAs, the longest open reading frames (ORFs) were extracted using TransDecoder (v5.5.0) [12] with the “TransDecoder.LongOrfs” command. Candidate mRNAs with ORFs longer than 200 bp were retained as novel mRNAs. Coding sequences (CDS) and protein sequences for these novel mRNAs were generated using the “TransDecoder.Predict” function.

**Identification of alternative splicing**

Alternative splicing events were identified using rMATs [14] on BAM files from RNA-seq data. Each sample’s BAM file was compared to the reference annotation, generating result files with the “rmats” suffix. These files were aggregated to identify splicing events. High-confidence alternative splicing events between tissues were identified by retaining events with an IncLevel variance greater than 0.1 and at least 20 mapped reads.

**SNP and InDel calling**

Aligned RNA-Seq reads were deduplicated using sambamba (v4.6.0.0) [15]. To enhance detection of genomic variation, BAM files from 10 tissues (representing different systems) for each species or breed were merged using Samtools (v1.15) [16], resulting in a single merged BAM file per species.

For each merged BAM file, the SplitNCigarReads function of GATK (v4.6.0.0) [17] was used to remove intronic regions. SNPs and InDels were called using GATK’s HaplotypeCallerSpark, and raw variants were filtered using GATK’s SelectVariants with the parameters “QUAL < 30.0 | QD < 2.0 | MQ < 30.0”. Annotated SNPs and InDels were produced using snpEff (v5.2a) [18].

**REFERENCES**

1. Brown, Michael C, and Christopher E Turner. 2004. “Paxillin: adapting to change.” *Physiological Reviews* 84: 1315-1339. <https://doi.org/10.1152/physrev.00002.2004>.

2. Zhu, Jiang, Fuhong He, Songnian Hu and Jun Yu. 2008. “On the nature of human housekeeping genes.” *Trends in Genetics* 24: 481-484. <https://doi.org/10.1016/j.tig.2008.08.004>.

3. Yanai, Itai, Hila Benjamin, Michael Shmoish, Vered Chalifa-Caspi, Maxim Shklar, Ron Ophir, Bar-Even, et al. 2005. “Genome-wide midrange transcription profiles reveal expression level relationships in human tissue specification.” *Bioinformatics* 21: 650-659. <https://doi.org/10.1093/bioinformatics/bti042>.

4. Pertea, Mihaela, Geo M Pertea, Corina M Antonescu, Tsung-Cheng Chang, Joshua T Mendell and Steven L Salzberg. 2015. “StringTie enables improved reconstruction of a transcriptome from RNA-seq reads.” *Nature Biotechnology* 33: 290-295. <https://doi.org/10.1038/nbt.3122>.

5. Niknafs, S Yashar, Balaji Pandian, Hariharan K Iyer, Arul M Chinnaiyan and Matthew K Iyer. 2017. “TACO produces robust multisample transcriptome assemblies from RNA-seq.” *Nature Methods* 14: 68-70. <https://doi.org/10.1038/nmeth.4078>.

6. Pertea, Geo and Pertea Mihaela. 2020. “GFF utilities: GffRead and GffCompare.” *F1000Research* 9: 304. https://doi.org/10.12688/f1000research.23297.2.

7. Trapnell, Cole, Brian A Williams, Geo Pertea, Ali Mortazavi, Gordon Kwan, Marijke J van Baren, Steven L Salzberg, *et al*. 2010. “Transcript assembly and quantification by RNA-Seq reveals unannotated transcripts and isoform switching during cell differentiation.” *Nature Biotechnology* 28: 511-515. <https://doi.org/10.1038/nbt.1621>.

8. Camacho, Christiam, George Coulouris, Vahram Avagyan, Ning Ma, Jason Papadopoulos, Kevin Bealer and Thomas L Madden. 2009. “BLAST+: architecture and applications.” *BMC Bioinformatics* 10: 421. <https://doi.org/10.1186/1471-2105-10-421>.

9. Kalvari, Ioanna, Eric P Nawrocki, Nancy Ontiveros-Palacios, Joanna Argasinska, Kevin Lamkiewicz, Manja Marz, Sam Griffiths-Jones, *et al*. 2020. “Rfam 14: expanded coverage of metagenomic, viral and microRNA families.” *Nucleic Acids Research* 49: D192-D200. https://doi.org/0.1093/nar/gkaa1047.

10. Kang, Yu-Jian, De-Chang Yang, Lei Kong, Mei Hou, Yu-Qi Meng, Liping Wei and Ge Gao. 2017. “CPC2: a fast and accurate coding potential calculator based on sequence intrinsic features.” *Nucleic Acids Research* 45: W12-W16. https://doi.org/10.1093/nar/gkaa104.

11. Li, Aimin, Haotian Zhou, Siqi Xiong, Junhuai Li, Saurav Mallik, Rong Fei, Yajun Liu, *et al*. 2024. “PLEKv2: predicting lncRNAs and mRNAs based on intrinsic sequence features and the coding-net model.” *BMC Genomics* 25: 756. <https://doi.org/10.1186/s12864-024-10662-y>.

12. Sun, Liang, Haitao Luo, Dechao Bu, Guoguang Zhao, Kuntao Yu, Changhai Zhang, Yuanning Liu, *et al*. 2013. “Utilizing sequence intrinsic composition to classify protein-coding and long non-coding transcripts.” *Nucleic Acids Research* 41: e166. https://doi.org/10.1093/nar/gkt646.

13. Wang, Yuanyuan, Zhijie Xie, Eric Kutschera, Jenea I Adams, Kathryn E. Kadash-Edmondson, Yi Xing. 2024. “rMATS-turbo: an efficient and flexible computational tool for alternative splicing analysis of large-scale RNA-seq data.” *Nature Protocols* 19: 1083-1104. <https://doi.org/10.1038/s41596-023-00944-2>.

14. Tarasov, Artem, Albert J Vilella, Edwin Cuppen, Isaac J Nijman, Pjotr Prins. 2015. “Sambamba: fast processing of NGS alignment formats.” *Bioinformatics* 31: 2032–2034. <https://doi.org/10.1093/bioinformatics/btv098>.

15. Li, Heng, Bob Handsaker, Alec Wysoker, Tim Fennell, Jue Ruan, Nils Homer, Gabor Marth, *et al*. 2009. “The Sequence Alignment/Map format and SAMtools.” *Bioinformatics* 25: 2078–2079. <https://doi.org/10.1093/bioinformatics/btp352>.

16. McKenna, Aaron, Matthew Hanna, Eric Banks, Andrey Sivachenko, Kristian Cibulskis, Andrew Kernytsky, Kiran Garimella, *et al*. 2010. “The Genome Analysis Toolkit: a MapReduce framework for analyzing next-generation DNA sequencing data.” *Genome Research* 20: 1297-1303. <https://doi.org/10.1101/gr.107524.110>.

17. Cingolani, Pablo, Adrian Platts, Le Lily Wang, Melissa Coon, Tung Nguyen, Luan Wang, Susan J Land, *et al*. 2012. “A program for annotating and predicting the effects of single nucleotide polymorphisms, SnpEff: SNPs in the genome of Drosophila melanogaster strain w1118; iso-2; iso-3.” *Fly(Austin)* 6: 80-92. https://doi.org/10.4161/fly.19695.

**SUPPLEMENTARY FIGURES**


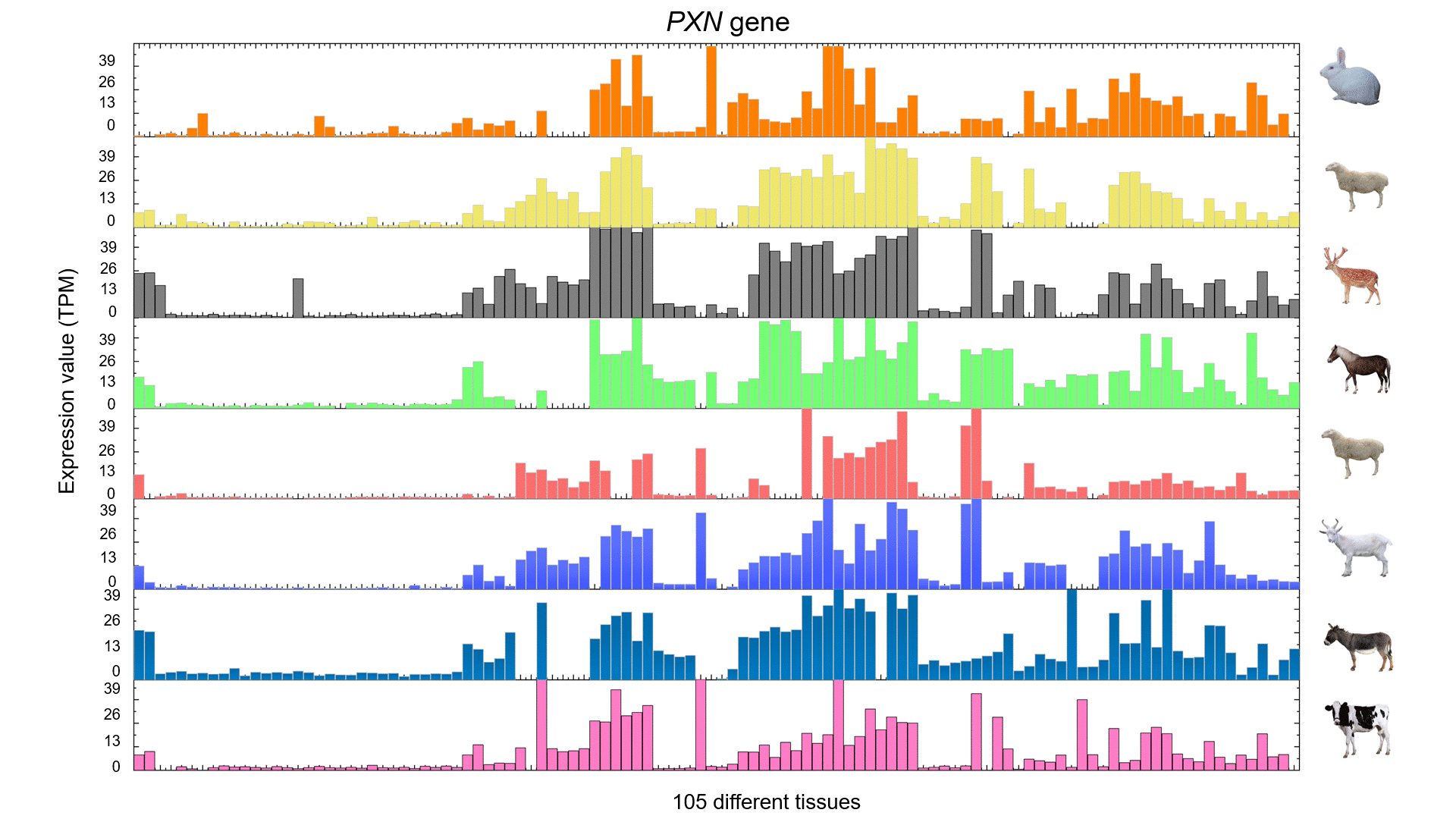


**Figure S1 An example showing the expression of *paxillin* gene between the seven species.**


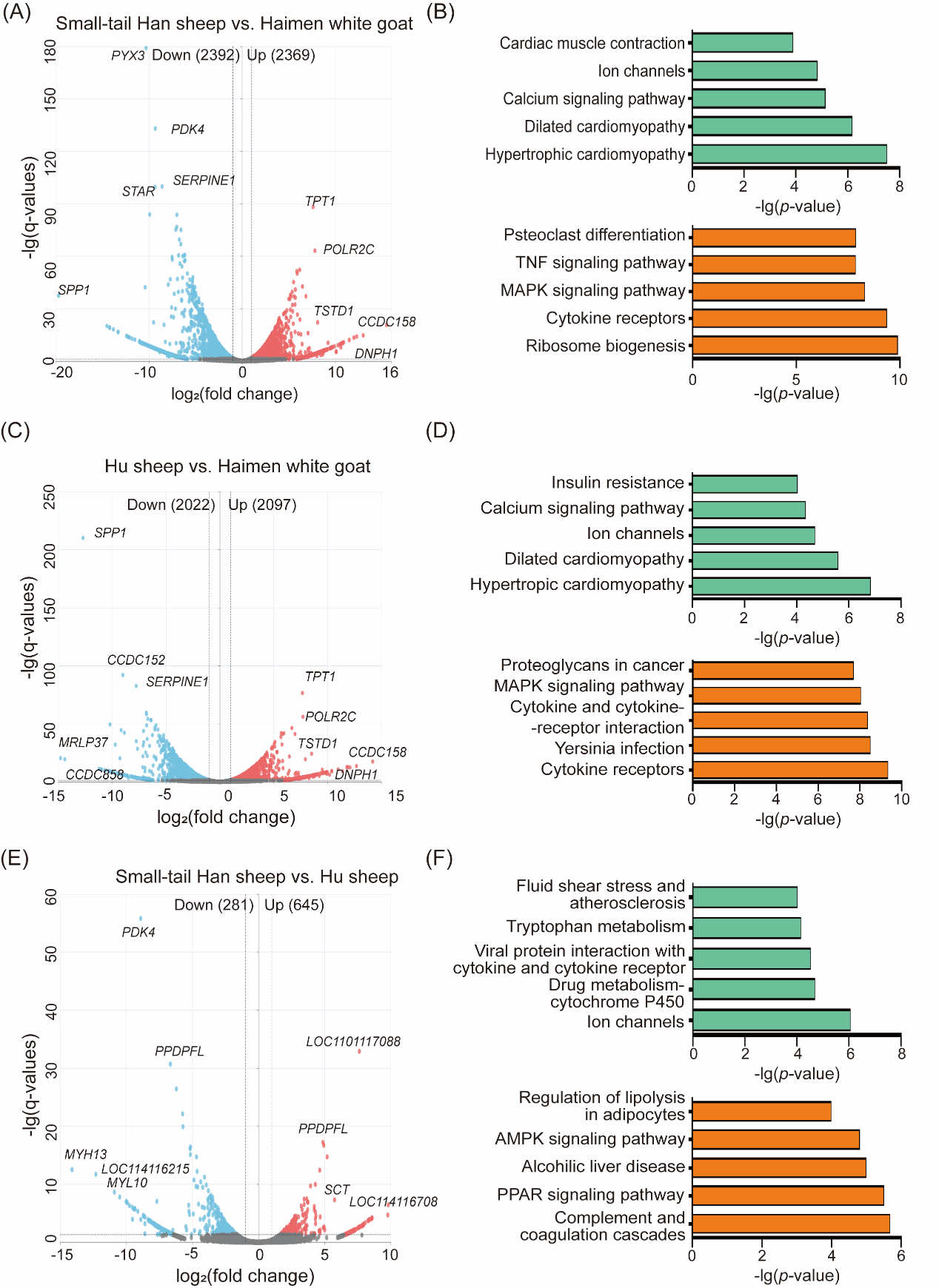


**Figure S2 Comparison of gene expression in the *longissimus dorsi* of sheep and goats.** (A) volcano plots of the differentially expressed genes (DEGs) in the *longissimus dorsi* between small-tail Han sheep and Haimen white goat, (C) Hu sheep and Haimen white goat, and (E) small-tail Han sheep and Hu sheep (red dots represent DEGs that are expressed more in the second species/breed, blue dots represent DEGs that are expressed less in the second species/breed). (B, D, and F) show Kyoto Encyclopedia of Genes and Genomes enrichments of the DEGs shown in red on the volcano plot (green bars) and the DEGs shown in blue on the volcano plot (orange bars).


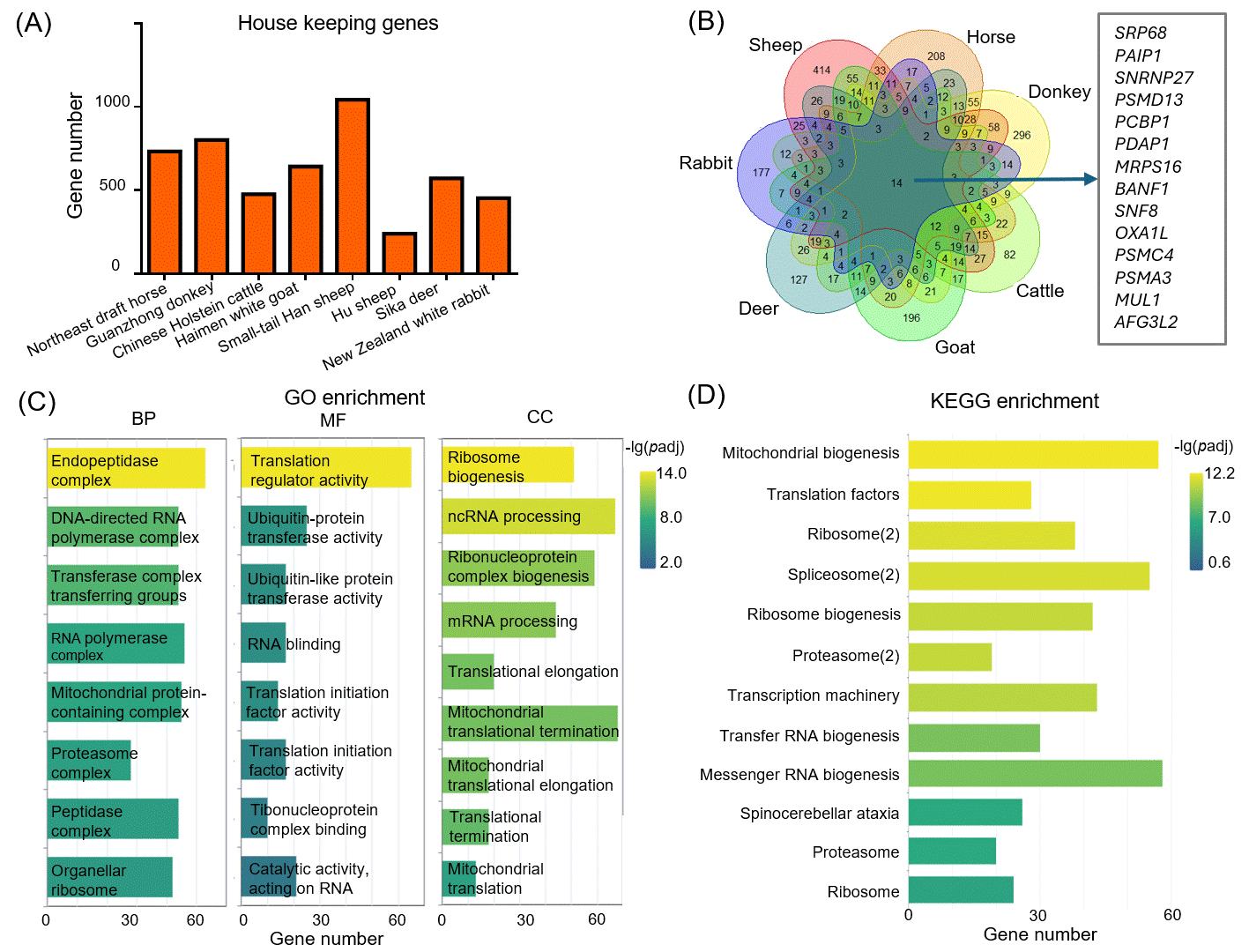


**Figure S3 An overview of the pattern of expression and functional enrichments of the housekeeping genes for each of eight breeds of herbivores.** (A) The number of the housekeeping genes (HKGs) in each of the eight species/breeds of herbivore, (B) co-expressed and individually expressed HKGs between the seven species, (C) GO enrichment of HKGs in Hu sheep for biological process (BP), molecular function (MF), and cellular component (CC), (D) Kyoto Encyclopedia of Genes and Genomes enrichment of the HKGs in Hu sheep.
